# Modulation of Stat-1 in Human Macrophages Infected with Different Species of Intracellular Pathogenic Bacteria

**DOI:** 10.1155/2016/5086928

**Published:** 2016-06-29

**Authors:** Giuditta Fiorella Schiavano, Sabrina Dominici, Laura Rinaldi, Alfonsina Mariarosaria Cangiano, Giorgio Brandi, Mauro Magnani

**Affiliations:** ^1^Department of Biomolecular Sciences, Section of Toxicological, Hygienic and Environmental Sciences, University of Urbino Carlo Bo, Via S. Chiara 27, 61029 Urbino, Italy; ^2^Department of Biomolecular Sciences, Section of Biochemistry and Molecular Biology, University of Urbino Carlo Bo, Via Saffi 2, 61029 Urbino, Italy

## Abstract

The infection of human macrophages by pathogenic bacteria induces different signaling pathways depending on the type of cellular receptors involved in the microorganism entry and on their mechanism(s) of survival and replication in the host cell. It was reported that Stat proteins play an important role in this process. In the present study, we investigate the changes in Stat-1 activation (phosphorylation in p-tyr_701_) after uptake of two Gram-positive (*Listeria monocytogenes* and* Staphylococcus aureus*) and two Gram-negative bacteria (*Salmonella typhimurium* and* Legionella pneumophila*) characterized by their varying abilities to enter, survive, and replicate in human macrophages. Comparing the results obtained with Gram-negative and Gram-positive bacteria, Stat-1 activation in macrophages does not seem to be related to LPS content. The p-tyr_701_Stat-1 expression levels were found to be independent of the internalized bacterial number and IFN-*γ* release. On the contrary, Jak/Stat-1 pathway activation only occurs when an active infection has been established in the host macrophage, and it is plausible that the differences in the expression levels of p-tyr_701_Stat-1 could be due to different survival mechanisms or to differences in bacteria life cycles within macrophages.

## 1. Introduction

Intracellular bacterial pathogens are a group of microorganisms that have developed many abilities to survive and replicate in mammalian cells [1]; thus, they are protected from the defense mechanisms of the host, such as specific antibodies and complement, making their exposition to antimicrobial agents more difficult. As a consequence, they are one of the major causes of global morbidity and mortality.

Although any type of tissue cells potentially serves as a habitat, for most intracellular bacteria, the macrophage represents the typical host cell. Indeed, although macrophages contribute to the first line of defense against infection through phagocytosis, paradoxically, in host-pathogen interactions, they can also become a reservoir for several pathogenic bacteria. This occurs because these pathogens have evolved to reside within the hostile environment of macrophages and are able to avoid host cell death.

When macrophages make contact with bacteria, several signal-transduction pathways are activated [2] stimulating phagocytosis. This cellular function is extremely complex, and no single model can fully account for the diverse kind of microorganisms that enter macrophages. In fact, different bacteria-recognition receptors induce different signaling pathways according to the type of cellular receptors called into play by microorganism entry.

The signal transducer and activator of transcription 1 (Stat-1) is an indispensable component of the cellular response to interferons (IFNs) during the immune reaction to pathogens. In fact, interferons employ receptor-associated Janus kinases (Jaks) to activate Stats by tyrosine phosphorylation [3].

Recent findings suggest that Stat proteins play an important role in bacterial infections and in the activation of the innate immune response [4]. In fact, it is known that the Jak/Stat-1 pathway constitutes one of the main ways to activate macrophages, upregulating the expression of many different genes associated with the secondary cell responses leading to macrophage activation or apoptosis [5]. In a previous study [6] we demonstrated that* Mycobacterium avium* infection induces Stat-1 hyperactivation for a considerable period and that it is important for macrophage survival after the establishment of an infection. Hence, it represents a potential target for drugs lowering phosphorylate Stat-1 expression levels.

In a subsequent study [7] we showed that internalization of heat-killed* M. avium* or inert particles induced a fast and transient activity of the Jak/Stat-1 pathway when internalized by Fc*γ* receptor engagement, probably as a preventive defense mechanism. However, a persistent Stat-1 pathway activation occurs only when an intracellular* M. avium* infection is established in macrophages.

Bacteria can be classified into two major groups, Gram-positive and Gram-negative, and their classification depends on staining determined by different cell wall components. In Gram-negative bacteria there is a strong presence of lipopolysaccharides (LPSs). LPSs are a potent macrophage-activating stimulus inducing the expression of several genes necessary for the execution of host defense function [8].

Interestingly, LPS and IFN are known to induce expression of a common set of genes in sensitive cell types such as macrophages. Although Stat-1 appears to play an essential role in various forms of innate immunity, its role in LPS-inducible gene expression is not fully understood [9]. Thus, since phagocytosis involves the recognition and binding of bacteria by receptors on the cell surface, Stat-1 activation could vary according to the type of bacteria involved in the infection: Gram-positive or Gram-negative. In the present study, we therefore selected both intracellular Gram-positive (*Listeria monocytogenes*,* Staphylococcus aureus*) and Gram-negative (*Salmonella typhimurium, Legionella pneumophila*) species responsible for infectious diseases in humans.

*L. monocytogenes*, a Gram-positive bacterium causing infections in immune-compromised patients and pregnant women [10], mainly recognizes TLR-2 and the TLR-2 dependent signaling as PI3K and Rac 1, and this mechanism is involved in the phagocytosis of* L. monocytogenes* by macrophages [11]. The bacterium is enclosed into the phagosome; however, it survives and escapes from the organelle producing a pore-forming toxin (listeriolysin-O) [12]. It has also been reported that* L. monocytogenes* can influence Stat-1 activity in a time-dependent manner [13].

*S. aureus* is one of the major human pathogens causing serious diseases such as nosocomial infections, impetigo, toxic shock syndrome, and bacteremia. After engulfment,* S. aureus* persists for several days inside macrophages using them as vehicles for the dissemination of the infection. The* S. aureus* then proliferate into the cytoplasm causing host cell lysis [14, 15]. Peptidoglycan and lipoproteins of* S. aureus* have been proposed to be such ligands for TLR2 as a cellular innate immune receptor, although the bacterium could be sensed by a receptor other than TLR2 [16, 17]. Other authors have shown that the phagocytosis of* S. aureus* occurs after binding on various receptors, including FcyRs and CR3, and contributes to the activation of MAPKs cascades [18].

*S. typhimurium,* an intracellular facultative pathogen responsible for gastroenteritis and other enteric diseases, invades and replicates within the host cell after binding on macrophage surfaces. During the initial contact with host cells, a group of effectors is delivered across the plasma membrane to modulate host signal transduction pathways, including the activation of Rho family GTPases, inducing actin rearrangements and the uptake of the bacteria into* Salmonella*-containing vacuoles (SCVs). By modifying SCV fusion using type III secreted effectors and other virulence factors, the bacteria are able to block SCV fusion with lysosomes [19, 20]. TLR4 plays an important role in invasive salmonellosis [21]. MAPK kinase (MEK) and ERK are activated by virulent* Salmonella* in the very early stages of LPS stimulation and macrophage infection [22].

*L. pneumophila* is an intracellular pathogen causing a severe and atypical pneumonia called Legionnaire's disease and the influenza-like illness called Pontiac fever.* L. pneumophila* invades macrophages using a bacterial surface protein, MOMP, that fixes complement component C3 to the surface of the parasite [23]. After internalization, the* Legionella*-containing vacuole evades transport to the lysosomal network and subsequent degradation.* L. pneumophila* remodels its compartment using a type of IVB secretion apparatus, and within this protected vacuole, the bacterium becomes acid tolerant, downregulates virulence factor expression, and establishes a replicative niche [24].

Highly specialized mechanisms have been evolved by intracellular bacteria to avoid or survive the hostile environment of the macrophage phagosome. These mechanisms culminate in the expression of inflammatory cytokine and chemokine genes as well as the regulation of endosomal trafficking [2].

In the present work, we investigated the changes in Stat-1 activity after the uptake of Gram-positive and Gram-negative bacteria with their varying abilities to enter, replicate, and survive in human macrophages.

## 2. Material and Methods

### 2.1. Bacteria

*S. typhimurium* (clinical isolate),* L. monocytogenes* (ATCC 9525), and* S. aureus* (clinical isolate) were initially grown overnight (16–18 hours) at 37°C in Tryptic Soy Broth (TSB). Samples were diluted 50-fold in fresh TSB and grown under aerobic conditions to an optical density (OD) corresponding to about 1 × 10^8^ cells per mL. Cultures were then collected by centrifugation at room temperature, washed once with PBS, and resuspended in RPMI 1640 medium supplemented with 10% (v/v) human pooled serum (AB).* L. pneumophila* (clinical isolate) were initially grown in buffered charcoal yeast extract (BCYE) agar for 72 h. Some colonies were then harvested and suspended in PBS to obtain an OD corresponding to approximatively 1 × 10^8^ bacteria per mL. They were then washed once and resuspended in RPMI 1640 medium supplemented with 10% serum AB. The bacterial number in each suspension was subsequently estimated by standard plate counts.

### 2.2. Macrophages

Monocyte-derived macrophages were prepared from leukocyte buffy coats obtained from healthy donors and purified as previously described [6]. Briefly, the peripheral blood mononuclear cells were isolated by Histopaque 1077 (Sigma Chemical Co.) gradient centrifugation and monocytes were separated from lymphocytes by adherence to plastic dishes, scraped, washed twice, and suspended in RPMI 1640 medium (cell culture tested) with 10% (v/v) inactivated fetal bovine serum and 1% antibiotics (v/v). Aliquots of 1 × 10^6^ cells were seeded in 35 mm Petri dishes and cultured at 37°C in 5% CO_2_ atmosphere for 10 days, at which time the monocytes had adhered to the plastic and matured into macrophages.

### 2.3. Phagocytosis Assay

Bacteria were suspended in RPMI 1640 medium supplemented with 10% heat inactivated human autologous (AB) serum to promote opsonization and subsequent internalization into phagocytic cells. Bacteria were then added to mature macrophages at a multiplicity of infection (MOI) of 25 : 1 (*S. aureus*/macrophage), 50 : 1 (*L. monocytogenes*/macrophage), or 100 : 1 (*L. pneumophila*/macrophage and* S. typhimurium*/macrophage) and incubated for 20 min (*S. typhimurium*), 60 min (*L. monocytogenes*), or 120 min (*S. aureus, L. pneumophila*) at 37°C in 5% CO_2_. Phagocytosis was stopped by putting the dishes on ice (time 0) and unbound or nonphagocytized bacteria were removed by an initial extensive washing with ice-cold HBSS. Any remaining extracellular bacteria were killed by bathing the macrophages for 2 h in RPMI 1640 medium containing 100 *μ*g/mL gentamycin. In pilot experiments, stepwise increasing concentrations of gentamicin (50–300 *μ*g/mL) gave increasingly efficient killing of external bacteria but constant yield of internalized bacteria upon cell lysis, suggesting that extracellular gentamicin did not compromise intracellular bacterial viability. To prevent extracellular growth of released bacteria the medium was then changed again with fresh media containing 10 *μ*g/mL gentamycin, and the cultures were incubated at 37°C in 5% CO_2_ for 3 days. The intracellular bacteria were evaluated after phagocytosis (time 0) and after 24 and 72 hours after phagocytosis. Briefly, infected macrophages were osmotically lysed with 1 mL of ice-cold distilled water, and the number of viable intracellular bacteria was assessed by the colony-forming units (CFU) assay from duplicate dishes on the appropriate agar medium. In parallel, the colony-forming assay was carried out in the culture media to exclude the presence of viable bacteria.

In a set of experiments, human macrophage cultures were exposed to* L. monocytogenes* and* S. typhimurium* as described above and incubated in the presence of 0.15 *μ*g/mL of anti-hIFN-*γ* antibody (clone 25718) (R&D System, Minneapolis, MN 55413) for 24, 48, and 72 hours at 37°C in 5% CO_2_. After incubation, macrophages were lysed to assess the number of intracellular CFU/mL by dilution plate counts on Plate Count Agar (PCA, Oxoid) and to evaluate Stat-1 pathway modulation.

### 2.4. Western Blot Assay for Stat-1 and p-tyr_701_Stat-1 Evaluation

After exposure to bacteria for 0, 24, 48, and 72 hours (experimental times), macrophages were lysed in lyses buffer (the lyses buffer consists of 50 mM Tris-HCl pH 7.8, 2% (w/v) SDS (sodium dodecyl sulfate), 5 mM EDTA (ethylenediaminetetraacetic acid), 109 mM NEM (N-ethylmaleimide), protease inhibitors: 2 mg/mL leupeptin, 2 mg/mL pepstatin, 4 mM AEBSF [4-(2-aminoethyl) benzenesulfonyl fluoride] and 1 mM PMSF (phenyl-methylsulphonylfluoride), and phosphatase inhibitors: 1 mM sodium orthovanadate and 1 mM of sodium fluoride). Cells lysates were boiled immediately for 5 min and centrifuged at 6,000 ×g for 13 min. The protein concentration of cell extracts was determined by the Lowry assay [25]. For the detection of p-tyr_701_Stat-1, ten micrograms of cell extracts was resolved on 7.5% SDS-PAGE and then blotted on Hybond-C Extra nitrocellulose membrane (Amersham Pharmacia Biotech, Italy) for 60 minutes at 100 V with a Bio-Rad Trans-Blot (Bio-Rad Laboratory, Germany) [26, 27]. For the immunoassay, membranes were treated with blocking solution [5% (w/v) nonfat dry milk dissolved in TBS (150 mM NaCl, 50 mM Tris, pH 7.5)] and maintained for 1 hour at room temperature. The specific immune-complexes were revealed after incubation with anti p-tyr_701_Stat-1 (cell signalling). An antiactin polyclonal antibody (Sigma Aldrich) was also used for the actin determination. The immune reactive bands were revealed after successive exposure to a horseradish peroxidase-conjugated anti rabbit IgG (Bio-Rad laboratory, Germany) followed by an enhanced chemiluminescence reaction (ECL, Amersham Pharmacia Biotech, Italy) [28]. Quantitative analysis was performed by a ChemiDoc System and Quantity One Program System (Bio-Rad Laboratory). The p-tyr_701_Stat-1 densitometric values were normalized on actin bands.

### 2.5. Statistical Analysis

The results are presented as means ± standard error (SEM) of at least 3 separate experiments. Statistical analyses were performed by the ANOVA type E* post hoc* test with the Graph Pad Prism 5 software. The significance of the data was evaluated on the control cells. A *P* value of less than 0.05 was determined to be statistically significant.

## 3. Results

### 3.1. Phagocytosis

In this study we selected two species of Gram-positive (*S. aureus* and* L. monocytogenes*) and two species of Gram-negative (*S. typhimurium* and* L. pneumophila*) intracellular bacteria to evaluate Stat-1 signaling pathway modulation after the phagocytosis of these pathogens by human macrophages. Macrophage cells were infected as reported in Section 2 and the intracellular number of viable bacteria was determined at each experimental time by enumeration of CFU.

**Figure 1:**
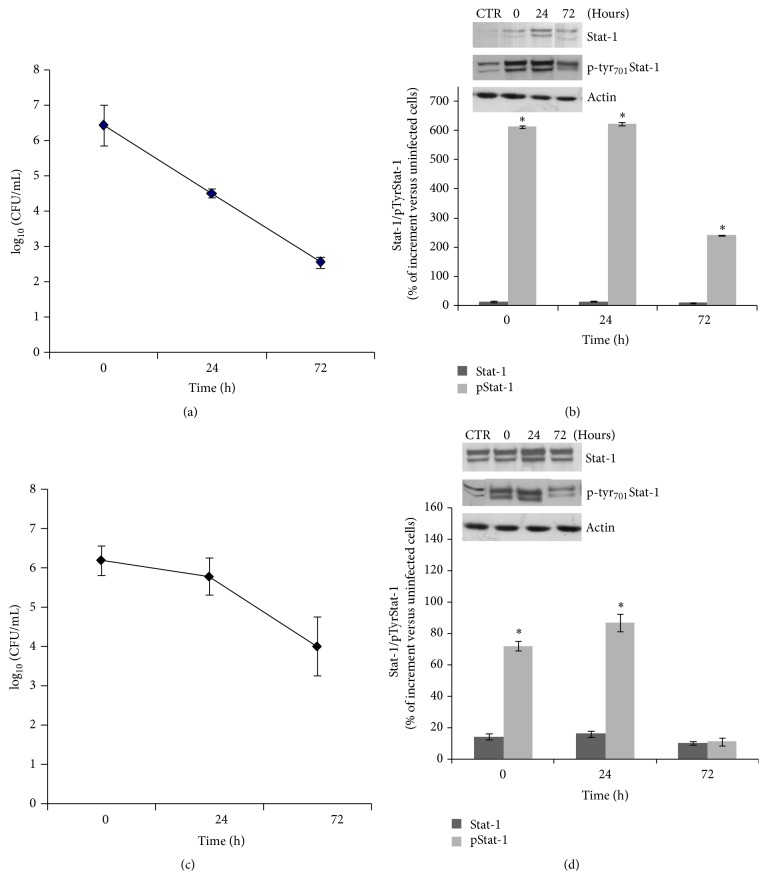
p-tyr_701_Stat-1 expression in human macrophages in response to Gram-positive bacterial infections. Ten-day-old macrophages were infected with* Staphylococcus aureus* (a, b) or* Listeria monocytogenes* (c, d). After removing extracellular microorganisms, cells were exposed for 30 min to gentamicin to kill noningested bacteria and then cultured for an additional 3 days in fresh medium containing gentamicin. At the indicated times, the intracellular viable bacteria were determined by enumeration of CFU in specific medium ((a) and (c)) and data are the mean of three independent experiments ± SEM. Stat-1 and p-tyr_701_Stat-1 expression levels were evaluated by Western blot assay at the same times ((b) and (d)). Data were normalized on untreated (control) cells as a percentage increase. ^*∗*^p-tyr_701_Stat-1 expression levels are significant compared to control cells.

**Figure 2:**
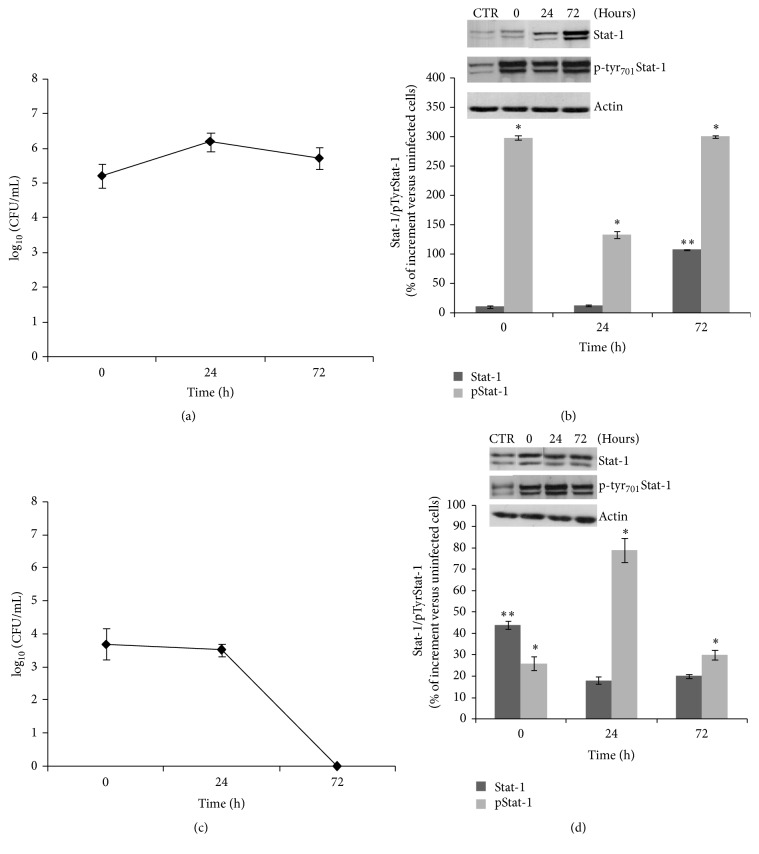
p-tyr_701_Stat-1 expression in human macrophages in response to Gram-negative bacterial infections. Ten-day-old macrophages were infected with* Salmonella typhimurium* (a, b) or* Legionella pneumophila* (c, d). After removing extracellular microorganisms, cells were exposed for 30 min to gentamicin to kill noningested bacteria and then cultured for an additional 3 days in fresh medium containing gentamicin. At the indicated time, the intracellular viable bacteria were determined by enumeration of CFU in specific medium ((a) and (c)), and data are the mean of three independent experiments ± SEM. Stat-1 and p-tyr_701_Stat-1 expression levels were evaluated by Western blot assay at the same times ((b) and (d)). Data were normalized on untreated (control) cells as a percentage increase. ^*∗*^p-tyr_701_Stat-1 expression levels are significant compared to control cells; ^*∗∗*^Stat-1 expression levels are significant compared to control cells.

As shown in Table 1 and Figure 2, 24 hours after phagocytosis the number of viable intracellular* S. typhimurium* increased up to 1 log and declined slightly (0.5 logs) after 72 hours from phagocytosis. In contrast, intracellular* S. aureus, L. monocytogenes,* and* L. pneumophila* decreased in a time-dependent manner after phagocytosis (Table 1, Figures 1(a), 1(b), and 2(b)). In particular, a marked decrease in viable intracellular bacteria was observed for* S. aureus* after 24 (up to −1.91 logs) and 72 (up to −3.87 logs) hours and for* L. pneumophila* after 72 hours (up to −3.81 logs), while the viable* L. monocytogenes* declined more gradually: up to −0.41 and −2.18 logs after 24 and 72 hours, respectively. Taken together these results confirm the differences among the four bacteria species in terms of their ability to survive intracellular killing in macrophages.

### 3.2. Stat-1/p-tyr_701_Stat-1 Pathway

In one set of experiments macrophages were infected with* S. aureus*,* L. monocytogenes, S. typhimurium*, and* L. pneumophila.* Then after 0, 24, and 72 hours, the levels of p-tyr_701_Stat-1 expression, used as a marker of Stat-1 pathway activity, were correlated with the amount of pathogens present inside the macrophage cells. In Figures 1 and 2 it is clearly shown that the Stat-1 pathway remains activated when the pathogen persists within the host cell. The p-tyr_701_Stat-1 expression levels do not always decrease in parallel with the number of bacteria in the cells.

In the case of Gram-positive infected macrophages (Figure 1), the p-tyr_701_Stat-1 expression levels increase immediately after phagocytosis reaching values up to 600% and 70% higher than values measured in uninfected cells for* S. aureus* (Figure 1(c)) and* L. monocytogenes* (Figure 1(d)), respectively. In these two cases, the p-tyr_701_Stat-1 levels were maintained for up to 24 hours before declining to much lower levels at 72 h in correlation with the elimination of the pathogen from the macrophage.

Gram-negative bacteria showed a similar trend to Gram-positive bacteria. Figure 2 shows macrophages infected with* S. typhimurium* (Figures 2(a) and 2(b)) and* L. pneumophila* (Figures 2(c) and 2(d)).* S. typhimurium* infected macrophages showed an increase in p-tyr_701_Stat-1 expression levels of up to 300% compared to control cells after phagocytosis (time 0). At 24 hours the increment of p-tyr_701_Stat-1 persisted, with values up to 135% higher than controls, while at 72 hours after infection, levels were once again found to be 300% higher than controls. In* L. pneumophila* infected macrophages, p-tyr_701_Stat-1 expression levels increased most significantly after 24 hours after phagocytosis (up to 80%), and in this case as well, these values were measured when pathogens were present in the host cell. Decreased levels of Stat-1 phosphorylation correlated with the elimination of the bacteria from the host.

### 3.3. IFN

**Figure 3:**
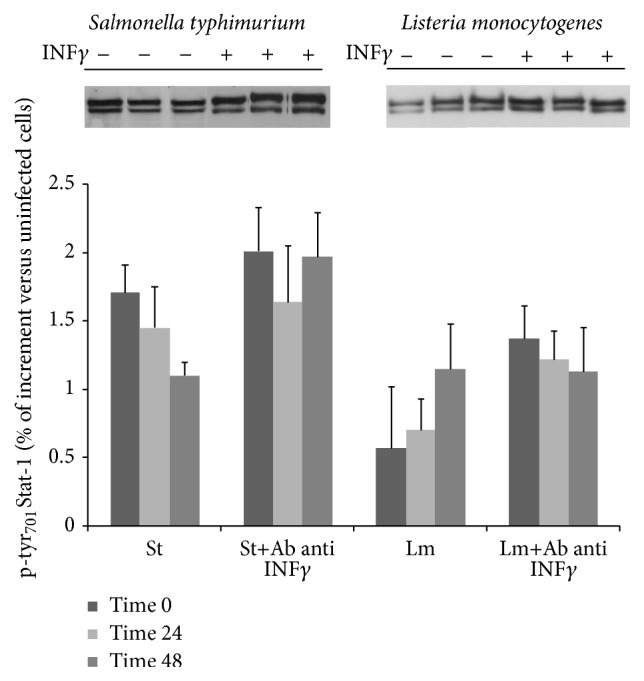
Densitometric analysis of p-tyr_701_Stat-1 expression levels in macrophages infected with* Salmonella typhimurium* (St) and* Listeria monocytogenes* (Lm). After infections, macrophages were cultured for 2 days in the absence or presence of 0.15 *μ*g/mL of anti-IFN-gamma antibody and Stat-1 and p-tyr_701_Stat-1 expression levels were evaluated by Western blot assay at the indicated times. Data were the mean of three different experiments. The differences of the p-tyr_701_Stat-1 expression levels between infected and infected cells after INF-gamma blocking were not significant.

Data reported in Figure 3 show that Stat-1/p-tyr_701_Stat-1 pathway activity is independent of INF*γ* release. In fact, macrophages infected with both* L. monocytogenes* and* S. typhimurium* showed high levels of expression of p-tyr_701_Stat-1 also in the presence of an antibody blocking INF*γ* release. The increased p-tyr_701_Stat-1 expression levels are consistent with the different ratio of replication of the two pathogens, respectively.

## 4. Discussion

Intracellular pathogens are responsible for much of the worldwide morbidity and mortality caused by infectious diseases. These pathogens are so dangerous because of their ability to survive and grow inside host macrophages [29]. Phagocytosis is a key process of defense in the host and begins with the binding of phagocytic receptors to their ligands on the surface of pathogens. Immunoglobulin receptors (FcRs) bind to bacteria opsonized by antibodies, while complement receptors (CRs) recognize bacteria opsonized by complement. In both cases, phagocytosis activates many signaling pathways to induce antibacterial effectors [30, 31]. However, the relative contribution of each signal transduction pathway to the fate of the internalized bacteria is not fully understood, and phagocytosis does not always lead to the eradication of microorganisms that survive and replicate in human macrophages.

Various findings suggest that Stat proteins play an important role in bacterial infections [5, 32]. In a previous work, we reported that live* M. avium* infection in macrophages kept the Jak/Stat-1 pathway active for a considerable period (more than 7 days), subsequent to the establishment of an intracellular infection and bacteria replication in the phagosome [7]. We believe that certain membrane receptors such as FcRs correlated with the Jak/Stat-1 signaling pathway activation and may be able to mediate bacilli phagocytosis or particle internalization in macrophages after opsonization. In particular, Stat-1 may play a pivotal role in the immune response and in the activation of macrophages, as demonstrated in knock-out mice [33, 34].

We have previously postulated that p-tyr_701_Stat-1 overexpression gives the infected cells a perfect target for cytotoxic drugs, such as fludarabine, which exert their action by inhibiting Stat-1 transcription [6]. It has also been postulated that Gram-negative and Gram-positive bacteria, while using different pattern recognition receptors, elicit signaling pathways that converge at the level of Stat-1 activation [13].

In the present study we investigated Stat-1 activation after the uptake of four bacterial species with varying abilities to enter, survive, and replicate in human macrophages. For this purpose we selected two Gram-positive bacteria (*L. monocytogenes* and* S. aureus*) and two Gram-negative bacteria (*S. typhimurium* and* L. pneumophila*) responsible for infectious diseases in humans.

It is known that the lipopolysaccharide (LPS) molecule, the major component of the outer membrane of Gram-negative bacteria, activates the Jak/Stat-1 pathway through LPS-induced CD40 expression [35]. In our previous works [6, 7], we postulated that all intracellular bacterial species induce notable activation of the Jak/Stat-1 pathway, although the extent of the activation varies according to the species. However, comparing the results obtained for Gram-negative and Gram-positive bacteria confirms that Stat-1 activation does not seem to be related to LPS content. The p-tyr_701_Stat-1 expression levels were found to be independent of the internalized bacterial number, which is consistent with what we observed in the our previous work on* M. avium* [7]. Thus, Jak/Stat-1 pathway activation occurs only when an active infection is established in the host, and it is plausible that the differences in the expression levels of p-tyr_701_Stat-1 could be due to different bacterial survival mechanisms or differences in their life cycles within macrophages. It is known that macrophages infected with intracellular pathogens release IFN-*γ* [36, 37], which potently activate macrophages to kill ingested organisms including bacteria. Moreover, IFN-*γ* efficiently stimulates overexpression of p-tyr_701_Stat-1. In this investigation, we showed that by blocking INF-*γ* release, p-tyr_701_Stat-1 expression levels in macrophages remained unchanged compared to values measured in untreated cells, suggesting that the Stat-1 activation was independent of IFN-*γ*.

## 5. Conclusion

Taken together, the findings of this work show a strong activation of the Stat-1 pathway in different intracellular pathogens without significant differences between Gram-positive and Gram-negative bacterial pathogens. The unchanged expression levels of p-tyr_701_Stat-1 after blocking the release of INF*γ* suggest that internalization and replication of pathogens in macrophages potentially activates the Stat-1 pathway by intracellular signaling. The exceptionally high expression levels of p-tyr_701_Stat-1 reconfirms Stat1 pathway activation as a marker to identify bacterial reservoirs. Such reservoirs could be eliminated after treatment with drugs blocking the Stat-1 pathway of Stat-1 phosphorylation. Further experiments will be carried out using animal disease models.

## Figures and Tables

**Table 1:** Variation in intracellular bacterial concentrations at different times after infection of human macrophages. Data are expressed as increment (+) or reduction (−) of log CFU ± SEM.

Bacterial species	Times	
	24 h	48 h
*Salmonella typhimurium*	+0.98 ± 0.09	+0.51 ± 0.13
*Listeria monocytogenes*	−0.41 ± 0.11	−2.18 ± 0.75
*Staphylococcus aureus*	−1.91 ± 0.47	−3.87 ± 0.53
*Legionella pneumophila*	−0.18 ± 0.40	−3.70 ± 0.40
